# Correlation Study of 3D Surface Roughness of Milled Surfaces with Laser Speckle Pattern

**DOI:** 10.3390/s22082842

**Published:** 2022-04-07

**Authors:** Suganandha Bharathi Jayabarathi, Mani Maran Ratnam

**Affiliations:** 1 Faculty of Engineering and Computer Technology, AIMST University, Semeling, Bedong 08100, Kedah, Malaysia; 2 School of Mechanical Engineering, Engineering Campus, Universiti Sains Malaysia, Nibong Tebal 14300, Penang, Malaysia

**Keywords:** surface roughness, laser speckle, milled surface

## Abstract

Current studies are focused on the correlation between characteristic features extracted from the laser speckle pattern of machined surfaces and 2D surface roughness parameters. Since milled surfaces are 3D in nature, 3D surface roughness parameters will provide a more accurate representation of the surface. Novelties of this work are: (1) an inexpensive laser pointer, which was used for presentation and was used without any spatial filtering setup for producing the laser speckle pattern; (2) a correlation study, which was conducted between characteristic features extracted from the speckle pattern and 3D surface roughness; and (3) the influence of angle of illumination, lens aperture size (*f*-number) and shutter speed on the correlation. A highest coefficient of determination of 0.8955 was obtained for the correlation between the gray level co-occurrence matrix descriptor, namely energy, and 3D surface roughness parameter, namely ten-point height S_10z_, at an illumination angle of 45°, *f*-number of 16 and shutter speed of 1/100 s.

## 1. Introduction

Surface roughness refers to finely spaced irregularities formed during the machining process [1]. Parameters such as the fit, wear resistance, fatigue strength, contact stiffness, vibration and noise of mechanical parts are related to surface roughness. These parameters will affect the service life and reliability of a product [2]. Therefore, surface roughness measurement is very important in manufacturing. Surface roughness is either measured using a contact method or a non-contact method. The contact method that is widely used in industry is the stylus probe. However, this method has several limitations, such as a lengthy measurement time and the fact that stylus accuracy depends on its tip radius and may not be able to accurately measure surfaces with crevices smaller than the stylus tip [3]. The stylus tip could also cause scratches on the surface of soft materials. Currently, available methods for determining 3D roughness parameters are mostly optical methods, which are non-contact methods, such as white light interferometers [4,5], the focus variation method [6] and confocal microscopy [7]. These methods have high requirements for the environment and have limited size and roughness measurement ranges. Machine visions have high measurement efficiency, the capacity to acquire large amounts of information, high measurement accuracy and good flexibility [2].

Some of the techniques that have been used for correlating the characteristic features with the surface roughness in the vision methods are statistical properties [8,9], wavelet transform [10], Tsallis Threshold [11], Neural Network [3], Gray Level Co-occurrence Matrix (GLCM) [12], Lacunarity [13] and spectral speckle correlation [14,15,16,17] and contrast [18,19]. Surface roughness parameters can be classified into profile roughness parameters and areal roughness parameters. The profile roughness parameters are also known as two-dimensional or 2D roughness parameters, and the areal roughness parameters are also known as three dimensional or 3D roughness parameters [8,20,21,22]. Current vision methods extract features to correlate with 2D roughness parameters [9,12,19,23,24], but machined surfaces are 3D in nature, and therefore, it is important to measure the 3D surface roughness parameters [8]. Most of the previous research work uses He-Ne laser or diode laser and spatial filters for cleaning the beam [9,19]. This setup is expensive. Laser pointers (<1 mW) class 2, which are used for presentation, will be used in the current work, which is inexpensive. Characteristic features extracted from the image of the laser speckle pattern of machined surfaces are based on the intensity of the pixels in the image. By varying the angle of illumination, interference between the scattered beams from the surface will vary, and this will result in a variation in intensity of the pixels. By adjusting the *f*-number, the amount of light that enters the camera can be controlled and, by increasing the *f*-number, the speckle size increases accordingly. In a similar manner, by increasing the shutter speed of the camera, the amount of light entering the camera can be controlled. Therefore, the variation of the angle of illumination of the laser beam, *f*-number and shutter speed affects the speckle pattern, and these, in turn, will affect the correlation of the characteristic features with 3D surface roughness. Hence, there is a need to study the effect of the angle of illumination of the laser beam, *f*-number and shutter speed on the correlation between the characteristic features with 3D surface roughness parameters. In addition, the aperture size and shutter speed of the camera play important roles in obtaining good contrast and brightness in the laser speckle image [25].

To the best knowledge of the authors, there is no work published on the following:

- Use of inexpensive laser pointers for producing the laser speckle pattern.
- A correlation study of the characteristic features extracted from the image of the laser speckle pattern of the milled surface and 3D surface roughness parameters, measured with commercial 3D metrology system.
- A study about the influence of the angle of illumination of a laser beam, *f*-number and the shutter speed of a camera setting on the correlation of 3D surface roughness parameters with the characteristic features extracted from the laser pattern image of the milled surface.

## 2. Materials and Methods

### 2.1. Sample Preparation

**Figure 1:**
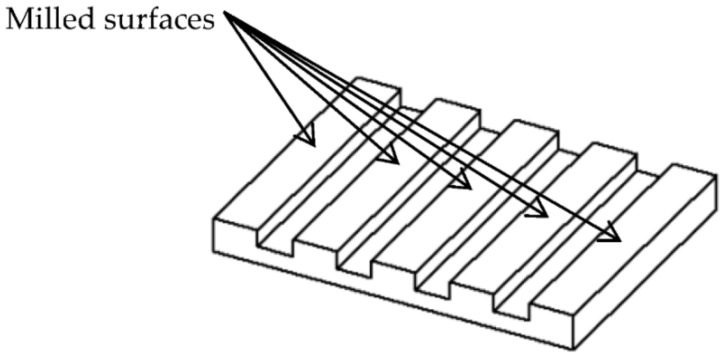
Isometric view of the sample.

**Figure 2:**
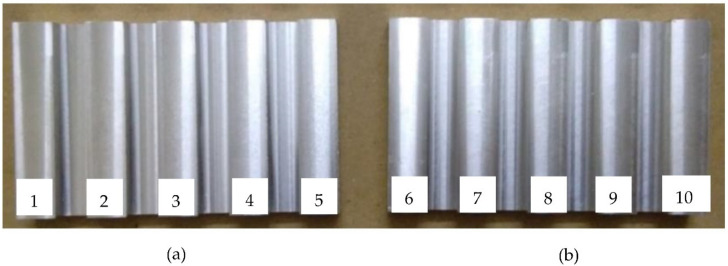
(**a**) Sample 1 and (**b**) sample 2 with surface numbering.

Two samples, each with five surfaces, were milled using a CNC 5 axis milling machine (DMU 40 monoBLOCK by Deckel Maho) with four flute endmill cutters of High-Speed Steel (HSS) with Ø12 mm. These reference samples are known as sample 1 and sample 2. Figure 1 shows the isometric view of one of the samples. Figure 2 shows the two real samples.

Each sample had 5 surfaces, which were machined at different feed rates and spindle speeds. Sample 1 surfaces were labeled as surface 1 to surface 5, whereas sample 2 surfaces were labeled as surface 6 to 10, as shown in Figure 2. Sample 1 was machined at feed rates of 120, 280, 440, 600 and 760 mm/min, at a spindle speed of 1000 rpm, and a depth of cut of 1 mm, whereas sample 2 was machined at feed rates of 120, 280, 440, 600 and 760 mm/min, at a spindle speed of 2500 rpm, and a depth of cut of 1 mm. Using an Alicona Infinite Focus Microscope by Bruker Alicona, which is a commercial 3D metrology system, the following 3D surface roughness parameters [26] were measured (cut-off wavelength of 141.58 µm and scan region of 708.32 µm by 537.35 µm setting on Alicona Infinite Focus) from the milled surfaces at 9 locations on each surface, and the average value is tabulated in Table 1:

1. Arithmetic Mean Height (*S_a_*)
2. Root-Mean-Square Height (*S_q_*)
3. Maximum Peak Height (*S_p_*)
4. Maximum Valley Depth (*S_v_*)
5. Maximum Height (*S_z_*)
6. Ten Point Height (*S_10z_*)
7. Skewness (*S_sk_*)
8. Kurtosis (*S_ku_*)
9. Root Mean Square Gradient (*S_dq_*)
10. Developed Interfacial area ratio (*S_dr_*)

### 2.2. Experimental Setup

**Figure 3:**
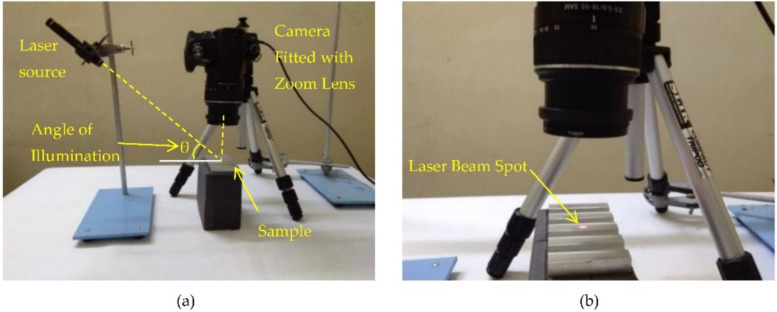
(**a**) Experimental setup and (**b**) close-up view of sample illuminated by laser beam.

**Figure 4:**
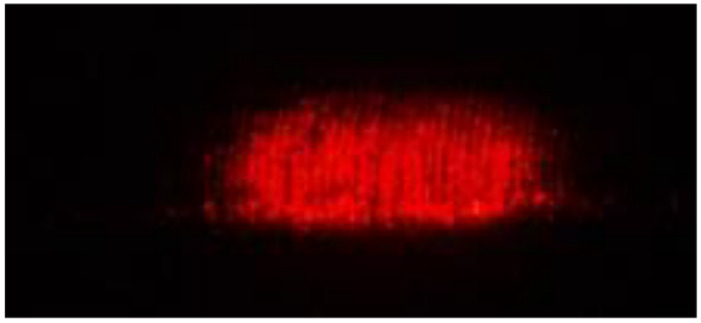
Laser speckle pattern.

Figure 3a shows the experimental setup. A laser beam from a commercial laser pointer (LX1 by Legamaster with a 5 mm diameter red laser dot, wavelength between 630 to 680 nm and a maximum output of less than 1 mW) was directed onto the sample at the desired angle, as shown in Figure 3a,b. The scattered beam underwent interference and formed a laser speckle pattern. Using a Sony Camera DSLR-A230 (image resolution of 3872 × 2592 pixels), fitted with an 18–55 mm smooth autofocus motor (SAM) Sony lens and close up +8 lens, a laser speckle pattern image of size 3872 × 2592 pixels was captured, as shown in Figure 4. The lens was set to manual focusing and a 55 mm focal length. To ensure no external lighting was available at the experiment setup, the experiment setup was covered with a black cloth.

The laser speckle patterns obtained from all ten milled surfaces were captured at various combinations of *f*-number and shutter speed settings of the camera. In this work, the illumination angles of the laser pointer used were 30°, 45° and 60°, the *f*-numbers used were 8, 16, 22 and 32, and the shutter speeds used were 1/50, 1/100, 1/200 and 1/400 s.

### 2.3. Characteristic Features Extraction

**Figure 5:**
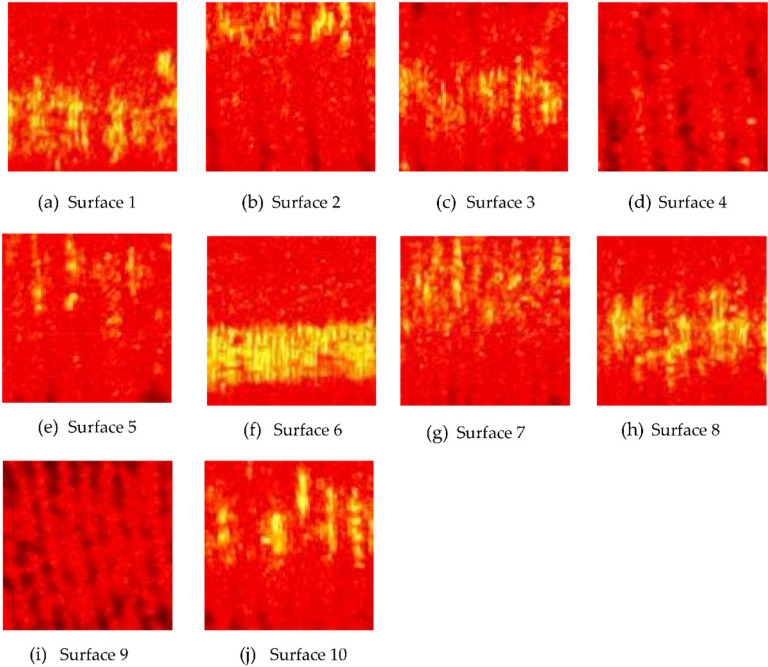
(**a**–**j**) are the cropped laser speckle pattern images for each surface at illumination angle of 45°, *f* number of 16 and shutter speed of 1/100 s.

The speckle patterns of all the ten surfaces were read into the MATLAB 2020b workspace, and a MATLAB program was developed for determining the centroid of each speckle pattern. Then, the speckle pattern was cropped to a size of 101 × 101 pixels with the centroid as the center of the cropped image, as shown in Figure 5a–j. A cropping size of 101× 101 was chosen to ensure that the cropping region was within the speckle pattern region.

The yellow color in the image, as shown in Figure 5, indicates a high-intensity region, and this high-intensity region is due to a specular highlight or reflected light caused by a tool mark [27]. The specular highlight will be along the center of the surface. Chatter marks due to vibration will cause an uneven depth of the tool mark, which will result in a higher intensity.

**Figure 6:**
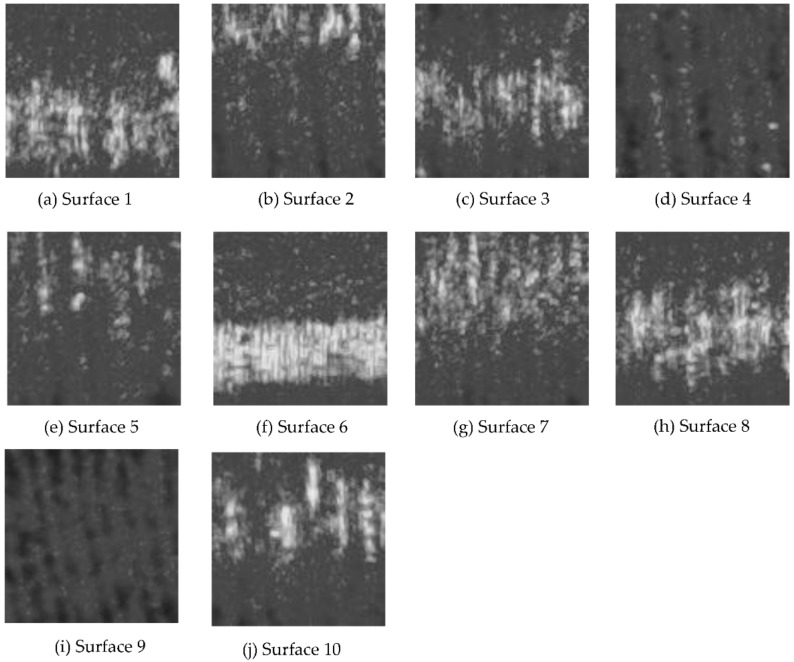
(**a**–**j**) are the grayscale images of the laser speckle pattern images for each surface at illumination angle of 45°, *f* number of 16 and shutter speed of 1/100 s.

**Figure 7:**
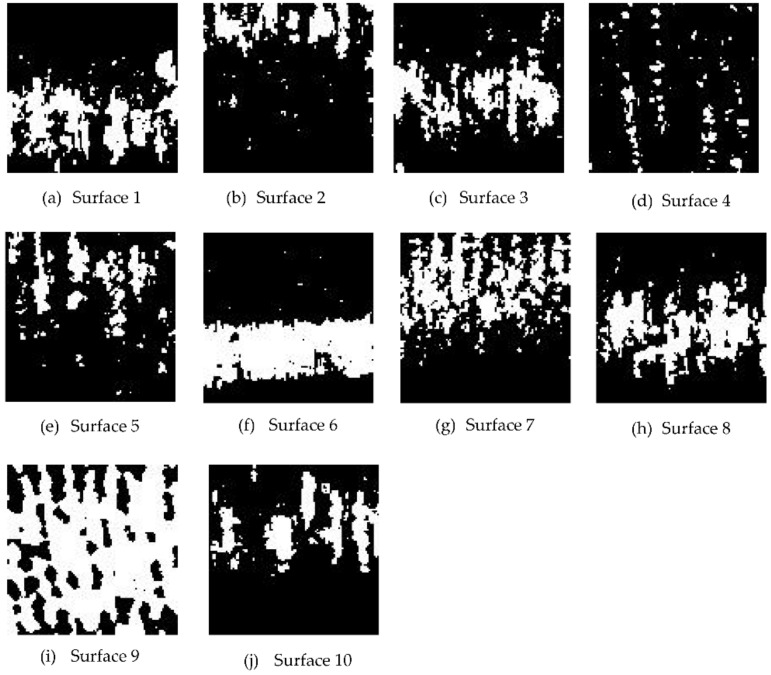
(**a**–**j**) are the binary images of the laser speckle pattern images for each surface at illumination angle of 45°, *f* number of 16 and shutter speed of 1/100 s.

Each image was then converted to grayscale, as shown in Figure 6. The grayscale image was not subjected to any filtering process in order to avoid loss of data caused by filtering. Characteristic features based on histograms, such as mean, standard deviation, energy, entropy and texture-based features, such as normalized roughness, and those based on gray level co-occurrence matrices (GLCM), such as maximum probability, correlation, contrast, energy, homogeneity and entropy, were extracted from the grayscale images. In order to differentiate the energy and entropy descriptors that are obtained from histograms and gray level co-occurrence matrices (GLCM), energy and entropy descriptors based on GLCM shall be addressed as energy (GLCM) and entropy (GLCM). The grayscale images were converted to binary, as shown in Figure 7. From the binary images, white-to-black pixel ratios were obtained as characteristic features. The extracted characteristic features were plotted against the 3D surface roughness, and the coefficients of determination were evaluated.

Equations (1)–(12) [28] were used to extract the characteristic features from the image as follows:

1. Histogram-based (statistical) features
  ∘ Mean
    Mean of gray value of the image *m* obtained from the original image *f*(*x,y*) of size *M* × *N* given by Equation (1).
    (1) $$m=\frac{1}{MN}\overset{M-1}{\underset{x=0}{\sum }}\overset{N-1}{\underset{y=0}{\sum }}f\left(x,y\right)$$
    where *f*(*x,y*) is the gray value of pixel at coordinates (*x,y*)
  ∘ Standard deviation
    Standard deviation *σ* of an image is given by Equation (2).
    (2) $$\sigma =\sqrt{\overset{L-1}{\underset{j=0}{\sum }}{\left(r_{j}-m\right)}^{2}p\left(r_{j}\right)}$$
    where:
    *r_j_* is the *j*th gray level.
    *L* is the total possible gray level value.
    *p*(*r_j_*) is the probability of occurrences of *r_j._*
    *m* is the mean of gray values of the image.
  ∘ Energy
    The energy descriptor, which is also known as uniformity, measures how pixel values are distributed along the gray level range and can be calculated for grayscale images using Equation (3).
    (3) $$energy=\overset{L-1}{\underset{j=0}{\sum }}{\left[p\left(r_{j}\right)\right]}^{2}$$
    where:
    *r_j_* is the *j*th gray level.
    *L* is total possible gray level value.
    *p*(*r_j_*) is the probability of occurrences of *r_j._*
  ∘ Entropy
    The entropy descriptor provides information about the complexity of the image, as given by Equation (4).
    (4) $$entropy=-\overset{L-1}{\underset{j=0}{\sum }}p\left(r_{j}\right)log_{2}\left[p\left(r_{j}\right)\right]$$
    where:
    *r_j_* is the *j*th gray level.
    *L* is total possible gray level value.
    *p*(*r_j_*) is the probability of occurrences of *r_j._*
2. Texture features
  ∘ Normalised descriptor of roughness *R*
    Normalised descriptor of roughness *R* is as given in Equation (5).
    (5) $$R=1-\frac{1}{1+\frac{\sigma^{2}}{{\left(L-1\right)}^{2}}}$$
    where:
    *σ*^2^ is variance.
    *L* is total possible gray level value
3. Gray level co-occurrence matrix (GLCM)
  The histogram-based texture descriptors do not provide any information about the spatial relationship among pixels. This information can be obtained using the gray level co-occurrence matrix (GLCM). The matrix holds the information about the number of times pixels with intensities *r_i_* and *r_j_* occur in the image *f*(*x,y*) in the position specified by the displacement vector *d =* (*d_x_,d_y_*) and orientation *θ*. In this work, the default values of the displacement vector and orientation, as in the MATLAB software, that is d = (0,1) and orientation of 0°, were used. The matrix is normalized as given in Equation (6).
  (6) $$N_{g}\;\left(i,j\right)=\frac{g\left(i,j\right)}{\sum_{i}\sum_{j}g\left(i,j\right)}$$
  where:
  *N_g_*(*i,j*) is the normalized gray level co-occurrence matrix.
  *g*(*i,j*) is the element of the gray level co-occurrence matrix.
  The following texture-based features are computed using a normalized GLCM, *N_g_*(*i,j*).
  ∘ Maximum Probability as given by Equation (7).
    (7) $$\mathrm{Maximum}\;\mathrm{probability}=\max\;N_{g}\left(i,j\right)$$
  ∘ Correlation as given by Equation (8).
    (8) $$\mathrm{Correlation}=\frac{\sum_{i}\sum_{j}\left(i-\mu_{i}\right)\left(j-\mu_{j}\right)N_{g}\left(i,j\right)}{\sigma_{i}\sigma_{j}}$$
    where:
    *µ_i_* is the mean of the row sums of *N_g_*(*i,j*).
    *µ_j_* is the mean of column sums of *N_g_*(*i,j*).
    *σ_i_* is the standard deviation of row sums of *N_g_*(*i,j*).
    *σ_j_* is the standard deviation of column sums of *N_g_*(*i,j*).
  ∘ Contrast as given by Equation (9).
    (9) $$\mathrm{Contrast}=\underset{i}{\sum }\underset{j}{\sum }{\left(i-j\right)}^{2}N_{g}\left(i,j\right)$$
  ∘ Energy as given by Equation (10).
    (10) $$\mathrm{Energy}=\underset{i}{\sum }\underset{j}{\sum }N_{g}^{2}\left(i,j\right)$$
  ∘ Homogeneity as given by Equation (11).
    (11) $$\mathrm{Homogeneity}=\underset{i}{\sum }\underset{j}{\sum }\frac{N_{g}\left(i,j\right)}{1+\left|i-j\right|}$$
  ∘ Entropy as given by Equation (12).
    (12) $$\mathrm{Entropy}=-\underset{i}{\sum }\underset{j}{\sum }N_{g}\left(i,j\right)log_{2}N_{g}\left(i,j\right)$$
4. From the binary image, the following characteristic features were extracted:
  ∘ Total white pixels to total black pixels ratio (W/B)

## 3. Results and Discussion

**Figure 8:**
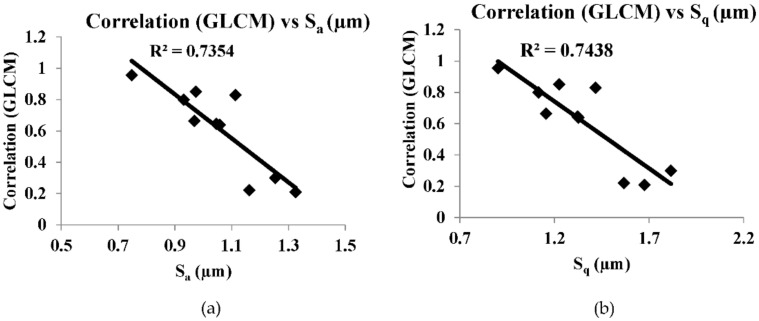
(**a**,**b**) are correlation plots between the characteristic features and roughness parameter with camera setting of *f*-number of 8, shutter speed 1/200 s and illumination angle of 30°.

**Figure 9:**
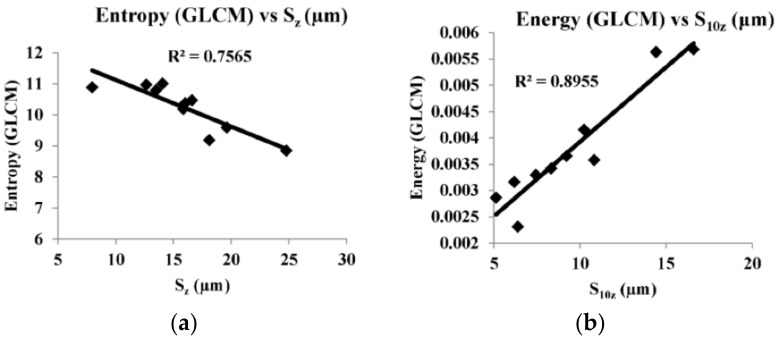
(**a**,**b**) are correlation plots between characteristic features and roughness parameter with camera setting of *f*-number of 8 and shutter speed 1/50 s and illumination angle of 45°.

**Figure 10:**
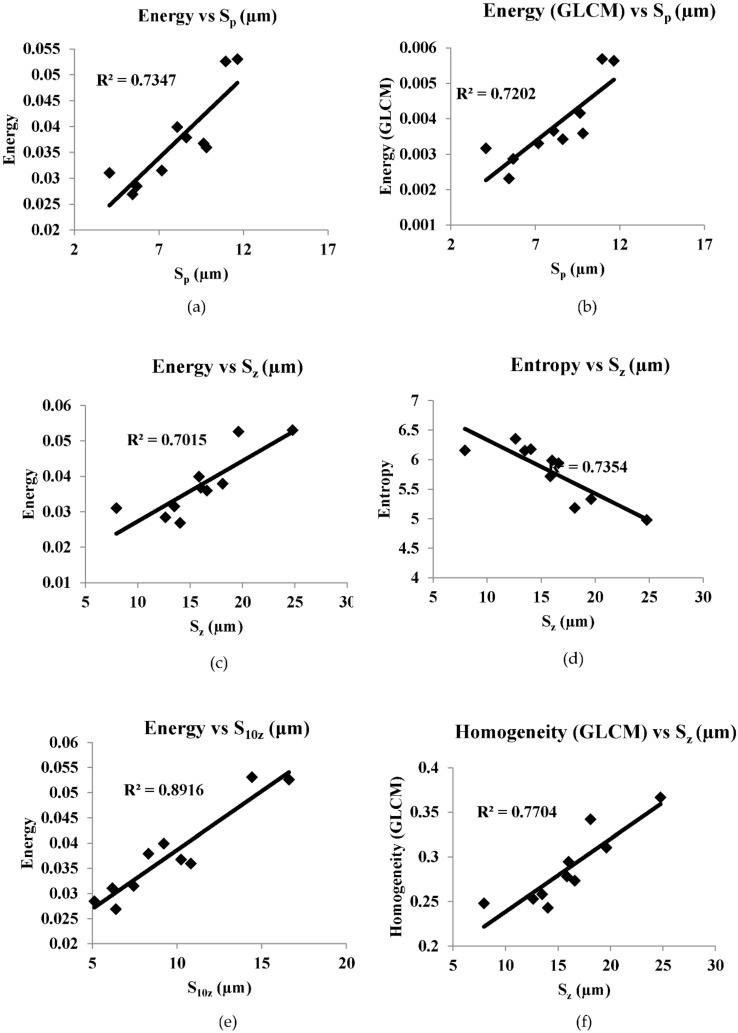

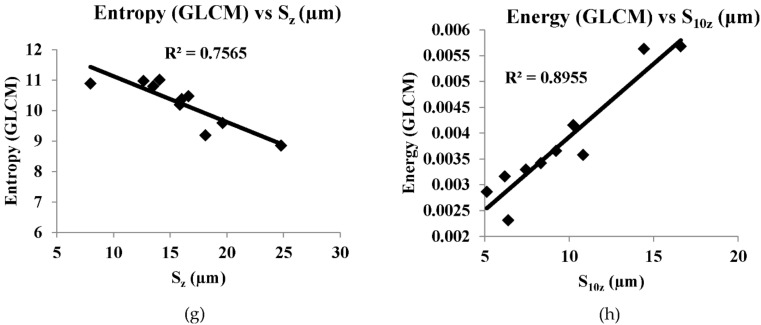
(**a**–**h**) are correlation plots between characteristic features and roughness parameter with camera setting of *f*-number of 16 and shutter speed 1/100 s and illumination angle of 45°.

**Figure 11:**
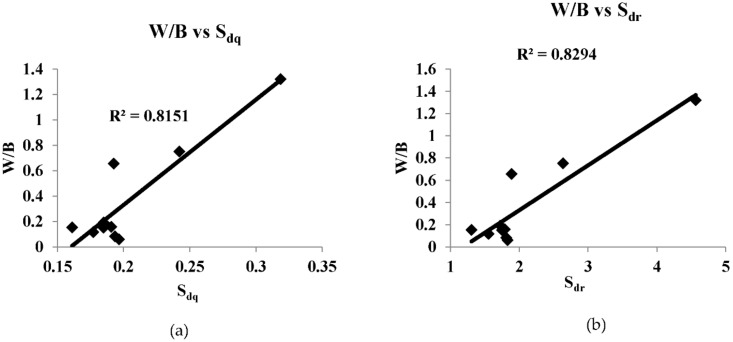
(**a**,**b**) are correlation plots between characteristic features and roughness parameter with camera setting of *f*-number of 22, shutter speed 1/100 s and illumination angle of 45°.

**Figure 12:**
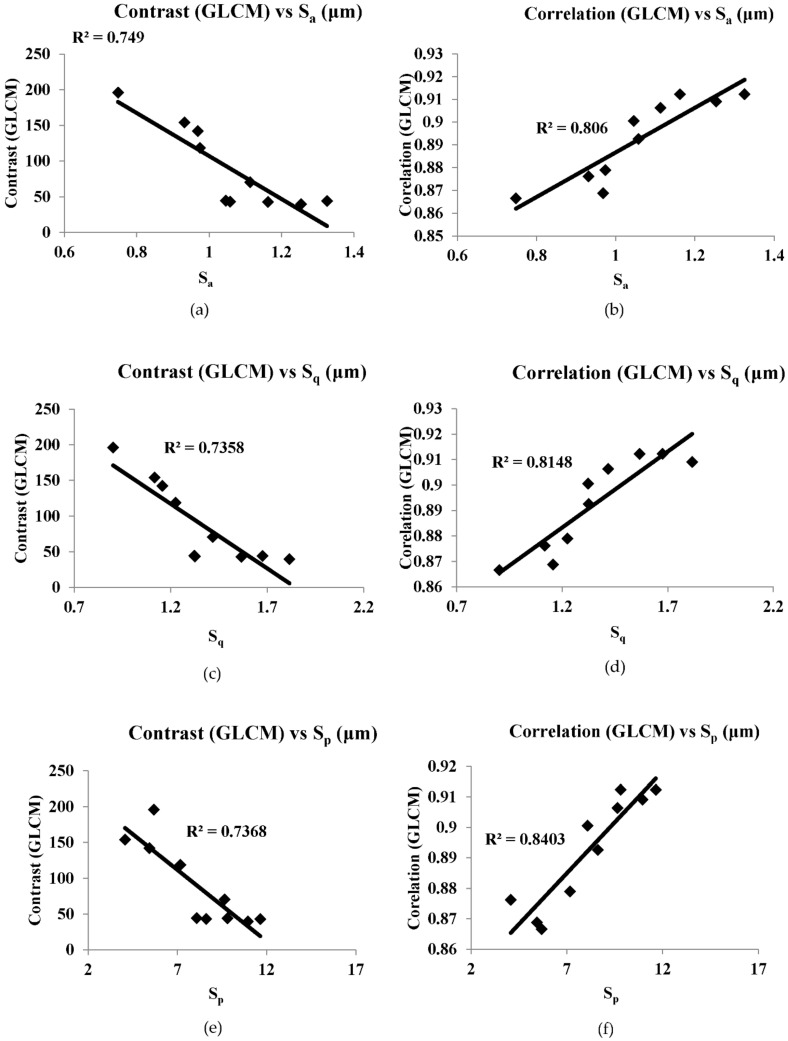

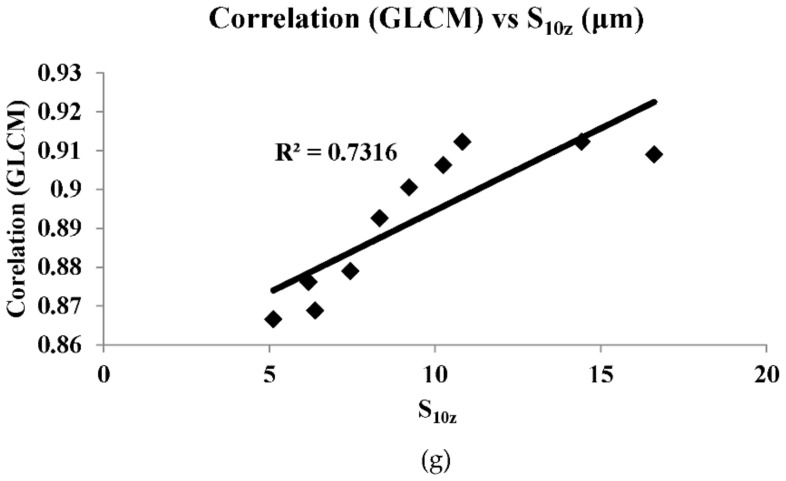
(**a**–**g**) are correlation plots between characteristic features and roughness parameter with camera setting of *f*-number of 22, shutter speed 1/200 s and illumination angle of 45°.

Figure 8, Figure 9, Figure 10, Figure 11 and Figure 12 are the plots between the characteristic features and 3D surface roughness with a coefficient of determination equal or above 0.7 for an illumination angle of 30° and 45°. Goh and Ratnam [8] used a linear fit for the correlations between characteristic features and 3D surface roughness parameters, and in this work the linear fit was used in the plot. Table 2 and Table 3 tabulates the coefficient of determination as mentioned in the plots.

The speckle pattern formed should not be very dark or too bright, as this will result in a poor correlation. Compared with illumination angles of 30°, illumination angles of 45° have more characteristic features, with a correlation of more than 0.7. This result is in line with other studies, which have reported that 2D surface roughness measurements were better at an incidence angle of 45° [29]. In addition to that, most of the GLCM characteristic features have a good correlation with the 3D surface roughness compared with the other characteristic features used in these works. Camera settings with an *f*-number of 16, a shutter speed of 1/100, an *f*-number of 22 and a shutter speed of 1/200 at an illumination angle of 45° have many characteristic features with good correlation with 3D surface roughness. A highest correlation of 0.8955 was obtained between energy (GLCM) and S_10z_ at an illumination angle of 45°, *f*-number 22 and shutter speed 1/100. There is no coefficient of determination of more than 0.7 in the case of illumination at an angle of 60°. At an illumination angle of 45°, better correlation between the characteristic features and 3D surface parameters compared to at illumination angles of 30° and 60° could be due to more scattered laser beams from the various secondary sources, which are at various peaks and valleys on the surface that represents the surface roughness, which undergoes interference normal to surface for an illumination angle of 45°. The camera is placed normal to the surface.

Out of the 21 plots shown in Figure 8, Figure 9, Figure 10, Figure 11 and Figure 12, it is found that 29% of the correlation above 0.7 involves correlation (GLCM), 14% are entropy (GLCM), energy and contrast (GLCM), 9.5% are energy (GLCM) and W/B and 5% are energy and homogeneity (GLCM). In a similar manner, 19% correlation above 0.7 involves *S_a_*, *S_q_*, *S_p_* and *S_z_*, 14% involves S_10z_ and 4.8% *S_dq_* and *S_dr_*.

Compared with an illumination angle of 30°, an illumination angle of 45° gives more characteristic features with a correlation more than 0.7. In addition, some characteristic features include correlations of more than 0.8 in the case of an illumination angle of 45° and no characteristic features, and a correlation of more than 0.8 in the case of an illumination angle of 30°. There is no correlation of more than 0.7 in the case of an illumination angle of 60°. On average, a shutter speed of 1/200 s produces better correlation (even if below 0.5) compared with other shutter speeds in the case of an illumination angle of 30°. In the case of illumination angles of 45 and 60°, on average, shutter speeds of 1/100 s and 1/200 s produce better correlation even if the correlation is below 0.5.

## 4. Conclusions

From the study, we can conclude that:

- An inexpensive laser pointer can be used for producing a laser speckle pattern.
- Good correlations between the characteristic features and 3D surface roughness were obtained.
- It was found that the angle of illumination, *f*-number and shutter speed combination affect the coefficient of determinations.

## 5. Recommendation for Future Work

- In this work, the authors limited their study using GLCM to displacement d = 1 and orientation $\theta =0^{{}^{\circ}}$. Future correlation studies should be carried out using GLCM for different combinations of displacement and orientation.
- A study should also be conducted on how the wavelength of a laser and the distance of the camera from the sample affect the correlation.

## Figures and Tables

**Table 1:** 3D surface roughness parameter for each milled surface.

Surface No.	Spindle Speed (rpm)	Feed Rate (mm/min)	Depth of Cut (mm)	*S_a_* (µm)	*S_q_* (µm)	*S_p_* (µm)	*S_v_* (µm)	*S_z_* (µm)	*S*_10z_ (µm)	*S_sk_*	*S_ku_*	*S_dq_*	*S_dr_* (%)
1	1000	120	1	0.931	1.117	4.070	3.889	7.959	6.187	−0.044	2.415	0.161	1.305
2	1000	280	1	1.046	1.322	8.081	7.774	15.855	9.217	0.281	3.398	0.177	1.553
3	1000	440	1	1.325	1.675	9.809	6.795	16.605	10.826	0.146	3.442	0.191	1.790
4	1000	600	1	1.162	1.567	11.639	13.136	24.775	14.423	0.275	6.054	0.242	2.636
5	1000	760	1	1.254	1.816	10.948	8.678	19.626	16.608	0.360	6.142	0.318	4.565
6	2500	120	1	0.748	0.902	5.681	6.960	12.641	5.122	0.335	2.473	0.185	1.722
7	2500	280	1	0.968	1.156	5.437	8.607	14.045	6.387	−0.177	6.528	0.185	1.749
8	2500	440	1	0.974	1.225	7.169	6.327	13.496	7.440	−0.124	4.374	0.196	1.830
9	2500	600	1	1.058	1.326	8.617	9.490	18.106	8.325	0.000	3.152	0.192	1.888
10	2500	760	1	1.113	1.417	9.640	6.377	16.016	10.246	0.257	3.715	0.193	1.811

**Table 2:** Tabulation of coefficient of determination value of more than 0.7 for illumination angle of 30°.

Correlation	R^2^	Camera Setting
Correlation (GLCM) vs. *S_a_*	0.7354	*f*-number 8 shutter speed 1/200 s
Correlation (GLCM) vs. *S_q_*	0.7438	

**Table 3:** Tabulation of coefficient of determination value of more than 0.7 for Illumination angle of 45°.

Correlation	*R* ^2^	Camera Setting
Entropy (GLCM) vs. *S_a_*	0.8208	*f*-number 8 shutter speed 1/50 s
Entropy (GLCM) vs. *S_q_*	0.7352	
Energy vs. *S_p_*	0.7347	*f*-number 16 shutter speed 1/100 s
Energy (GLCM) vs. *S_p_*	0.7202	
Energy vs. *S_z_*	0.7015	
Entropy vs. *S_z_*	0.7354	
Entropy (GLCM) vs. *S_z_*	0.7565	
Homogeneity (GLCM) vs. *S_z_*	0.7704	
Energy vs. *S*_10z_	0.8916	
Energy (GLCM) vs. *S*_10z_	0.8955	
W/B vs. *S_dq_*	0.8151	*f*-number 22 shutter speed 1/100 s
W/B vs. *S_dr_*	0.8294	
Contrast (GLCM) vs. *S_a_*	0.749	*f*-number 22 shutter speed 1/200 s
Correlation (GLCM) vs. *S_a_*	0.806	
Contrast (GLCM) vs. *S_q_*	0.7358	
Correlation (GLCM) vs. *S_q_*	0.8148	
Contrast (GLCM) vs. *S_p_*	0.7368	
Correlation (GLCM) vs. *S_p_*	0.8403	
Correlation (GLCM) vs. *S*_10z_	0.7316	

## Data Availability

Not applicable.
